# Systematic Review and Meta-Analysis of Detecting Galactomannan in Bronchoalveolar Lavage Fluid for Diagnosing Invasive *Aspergillosis*

**DOI:** 10.1371/journal.pone.0043347

**Published:** 2012-08-14

**Authors:** Mingxiang Zou, Lanhua Tang, Shushan Zhao, Zijin Zhao, Luyao Chen, Peng Chen, Zebing Huang, Jun Li, Lizhang Chen, Xuegong Fan

**Affiliations:** 1 Department of Clinical Laboratory, Xiangya Hospital, Central South University, Changsha, Hunan, China; 2 Eight-Year Program, Xiangya Hospital, Central South University, Changsha, Hunan, China; 3 Xiangya School of Medicine, Central South University, Changsha, Hunan, China; 4 Department of Infectious Diseases, Xiangya Hospital, Central South University, Changsha, Hunan, China; 5 School of Public Health, Central South University, Changsha, Hunan, China; Stony Brook University, United States of America

## Abstract

**Background:**

Bronchoalveolar lavage (BAL) galactomannan (GM) assay has been used for diagnosing invasive *aspergillosis* (IA). We aimed to derive a definitive estimate of the overall accuracy of BAL-GM for diagnosing IA.

**Methods and Results:**

We undertook a systematic review of thirty diagnostic studies that evaluated the BAL-GM assay for diagnosing IA. PubMed and CBM (China Biological Medicine Database) databasees were searched for relevant studies published in all languages up until Feb 2012. The pooled diagnostic odds ratio (DOR) and summary receiver operating characteristic (SROC) were constructed for each cutoff value. Additionally, pooled sensitivity (SEN), specificity (SPE), and positive and negative likelihood ratios (PLR and NLR, respectively) were calculated for summarizing overall test performance. Thirty studies were included in this meta-analysis. The summary estimates of pooled DOR, SEN, SPE, PLR, and NLR of the BAL-GM assay (cutoff value 0.5) for proven or probable IA were 52.7 (95% confidence interval (CI) 31.8–87.3), 0.87 (95% CI 0.79–0.92), 0.89 (95% CI 0.85–0.92), 8.0 (95% CI 5.7–11.1) and 0.15 (95% CI 0.10–0.23) respectively. The SROC was 0.94 (95% CI 0.92–0.96). Compared with cutoff value of 0.5, it has higher DOR, SPE and PLR, and similar SEN and NLR with cutoff value of 1.0, which indicated the optimal cutoff value might be 1.0. Compared with BAL-GM, serum GM has a lower SEN and higher SPE, while PCR displays a lower SEN and a similar SPE.

**Conclusion:**

With the optimal cutoff value of 1.0, the BAL-GM assay has higher SEN compared to PCR and serum GM test. It is a useful adjunct in the diagnosis of proven and probable IA.

## Introduction

Invasive *aspergillosis* (IA) is a potentially lethal infection, caused by *Aspergillus fumigatus* as well as other *Aspergillus* species which are widely distributed in soil and other organic matter[1], [2]. Currently, the rates of morbidity and mortality associated with IA infections are increasing as more and more number of patients undergo organ transplantation or allogeneic haematopoietic stem cell, and are treated with immunosuppressive agents [3], [4], [5].

Antifungal drugs, such as posaconazole, voriconazole, itraconazole and echinocandins, have greatly improved the therapeutic option for the treatment of IA[6]. Although the favorable clinical outcome in patients is largely influenced by the early initiation of effective treatment by antifungal drugs [7], early clinical diagnosing IA is still a critical problem and microbiological proof of IA is rarely feasible [3], [8]. Recently, GM, which is a heat-stable polysaccharide found in the fungal wall of most *Aspergillus* and *Penicillium* species[9], test has been developed to combat this issue[10], because diagnostic techniques using GM enzyme immunoassay performed on BALF have the potential to provide evidence of IA infection[11].

So far, several studies have assessed the diagnostic yield of GM testing in BAL for diagnosis of IA. A recent meta-analysis evaluated the quality of thirteen clinical studies that used the of BAL-GM test for diagnosing IA among patients, and concluded that, the BAL-GM test can be used as a major diagnostic method with excellent accuracy, however the BAL-GM test is not absolutely sensitive and specific[12]. Our research team performed a more systematic review of these and more recent clinical studies by meta-analysis to assess the accuracy of BAL-GM test method for diagnosing IA.

## Materials and Methods

### Literature Search

To identify eligible studies for this meta-analysis, two investigators (Zijin Zhao and Luyao Chen) searched the PubMed and CBM (China Biological Medicine Database) database in all languages which were published up to Feb 2012. The search strategy was based on Boolean combinations of the keywords ((Galactomannan or GM) AND (invasive aspergillosis or aspergillus) AND (bronchoalveolar lavage or pulmonary lavage)). As the review progressed, we improved the search strategies when necessary. All references cited in these studies were also reviewed to identify additional studies.

**Figure 1:**
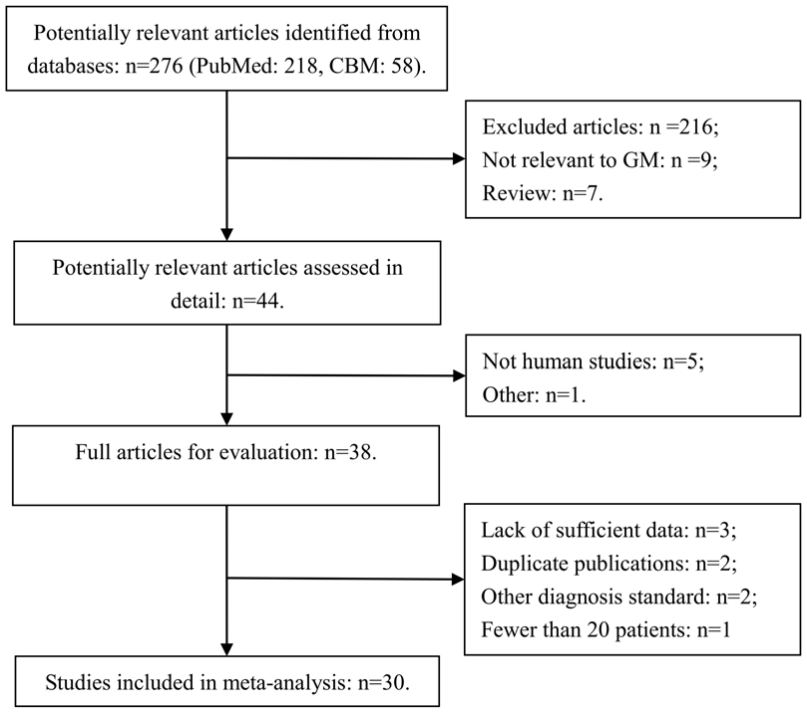
Flow diagram of study selection process.

**Figure 2:**
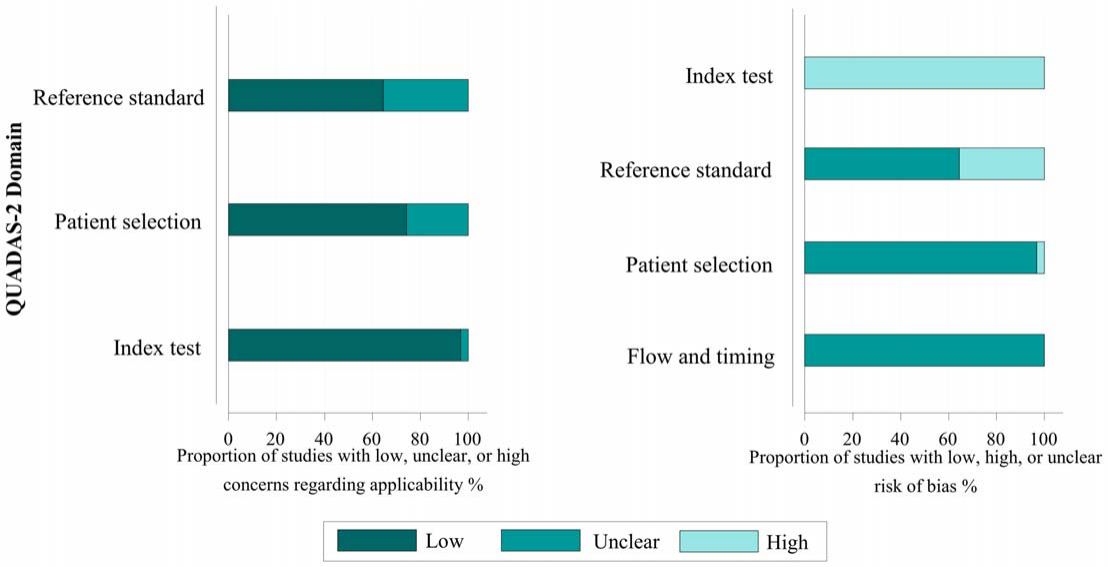
Overall quality assessment of included studies (QUADAS-2 tool).

### Inclusion/Exclusion Criteria

All relevant case-control or cohort studies were included, irrespective of publication status and language. In this meta-analysis, the following inclusive selection criteria were set and reviewed by two independent investigators: (1) full-text publications, (2) presenting original data for two-by-two tables: when multiple publications from a particular research group reported data from overlapping samples, the study reporting the largest dataset was included, (3) inclusion of patients according to the diagnosis standard of European Organization for Research and Treatment of Cancer/Mycoses Study Group (EORTC/MSG)[13], revised EORTC/MSG criteria14] or slight modification EORTC/MSG according to the research population as a reference standard. Results which were double-checked were arbitrated by a third investigator (Mingxiang Zou).

**Table 1:** Main Characteristics of Studies Included in the Meta-analysis of Diagnosis of IA using BAL-GM.

Study	Year	Region	Patient Population	Mean Age(year MD)	Male (%)	Study Design	Data Collection	Sampling Method	Diagnostic Standard	Diagnose
Bergeron *et al* [23]	2012	France	Adults with HM	52(10–78)	57.4	Cohort	Prospective	Consecutive	2008 EORTC/MSG	IPA
D'Haese *et al* [24]	2012	Belgium	Adults with MTHF	N/A	58.2*	Cohort	Retrospective	Consecutive	2008 EORTC/MSG	IPA
Acosta *et al* [*25*]	2011	Spain	Adults with MTHF	57.5±15.1*	60.0	Cohort	Prospective	Consecutive	2008 EORTC/MSG	IA
Hadrich *et al* [*26*]	2011	Tunisia	Any age with HM	37.6(1–84)	71.4	Case-control	Prospective	Consecutive	Modified 2008 EORTC/MSG	IA
Luong *et al* [*27*]	2011	America	Adults TR	54.7±10.8	62.5	Case-control	Retrospective	Unclear	2008 EORTC/MSG	IPA
Nguyen *et al* [*28*]	2011	America	Adults with MTHF	63*	73.1*	Cohort	Retrospective	Consecutive	2008 EORTC/MSG	IPA
Torelli *et al* [*29*]	2011	Italy	Patients with MTHF	N/A	N/A	Cohort	Prospective	NO	Modified 2008 EORTC/MSG	IA
Racil *et al* [*30*]	2011	Czech	Adults with HM	54 (18–79)*	65.7*	Cohort	Retrospective	Unclear	2008 EORTC/MSG	IPA
Leng *et al* [*31*]	2011	China	Adults with HM	N/A	NA	Cohort	Prospective	Unclear	Modified 2008 EORTC/MSG	IPA
Lin *et al* [*32*]	2011	China	Adults with MTHF	69.0±12.3	83.3	Case-control	Retrospective	Unclear	Modified 2008 EORTC/MSG	IPA
Bergeron *et al* [*33*]	2010	France	Adults with HM	43±20	63.6	Cohort	Retrospective	Consecutive	2008 EORTC/MSG	IPA
Park *et al* [*34*]	2010	Korea	Adults with MTHF	54 (16–74)	54.5	Cohort	Prospective	Consecutive	2008 EORTC/MSG	IPA
Hsu *et al* [*35*]	2010	Singapore	Adults with HM	35 (9–89)	80.0	Case-control	Prospective	Unclear	2008 EORTC/MSG	IPA
Danpornprasert *et al* [*36*]	2010	Thailand	Patients with MTHF	41 (16–75)	56.7	Cohort	Prospective	Unclear	Modified 2008 EORTC/MSG	IPA
Paugam *et al* [*37*]	2010	France	Adults with IC	N/A	N/A	Cohort	Retrospective	Unclear	Modified 2008 EORTC/MSG	IPA
Pasqualotto *et al* [*38*]	2010	Brazil	Patients with TR	55 (10–72)*	51.7*	Cohort	Prospective	NO	Modified 2008 EORTC/MSG	IA
Luong *et al* [*39*]	2010	Canada	Adults with HM	55*	65.3	Cohort	Retrospective	Unclear	2008 EORTC/MSG	IPA
Jin *et al* [*40*]	2010	China	Adults with MT	35(18–45)	60.0	Cohort	Retrospective	Unclear	Modified 2008 EORTC/MSG	IA
Maertens *et al* [*41*]	2009	Belgium	Adults with HM	53.6	N/A	Case-control	Retrospective	Unclear	2008 EORTC/MSG	IA
Desai *et al* [*42*]	2009	America	Children with MTHF	10.3	45.2	Cohort	Retrospective	Consecutive	Modified 2002 EORTC/MSG	IA
Frealle *et al* [*43*]	2009	France	Adults with MTHF	50.6(20–80)	64.0	Cohort	Retrospective	Unclear	2002 EORTC/MSG	IPA
Kimura *et al* [*44*]	2009	Japan	Adults with MTHF	74.5(49–79)	50.0	Case-control	Retrospective	Unclear	Modified 2002 EORTC/MSG	IPA
Meersseman *et al* [*45*]	2008	Belgium	Adults with MTHF	62	57.7	Cohort	Prospective	Consecutive	Modified 2002 EORTC/MSG	IA
Shahid *et al* [*46*]	2008	India	Adults with BC	58	91.3	Cohort	Prospective	Consecutive	Modified 2002 EORTC/MSG	IPA
Husain *et al* [*47*]	2008	Amercia	Adults TR	N/A	N/A	Cohort	Retrospective	Consecutive	2008 EORTC/MSG	IA
Clancy *et al* [*48*]	2007	Amercia	Adults TR	51.6(40–64)	100	Cohort	Retrospective	Consecutive	Modified 2002 EORTC/MSG	IPA
Nguyen *et al* [*49*]	2007	Amercia	Adults with MTHF	63.5(61–66)	100	Cohort	Prospective	Consecutive	Modified 2002 EORTC/MSG	IPA
Musher *et al* [*50*]	2004	Amercia	HSCT Adults	45.2	N/A	Case-control	Retrospective	Unclear	Modified 2002 EORTC/MSG	IPA
Becker-A *et al* [*51*]	2003	Netherlands	Adults with HM	49(18–79)	N/A	Cohort	Prospective	Unclear	Modified 2002 EORTC/MSG	IPA
Becker-B *et al* [*51*]	2003	Netherlands	Adults with HM	47(18–74)	N/A	Cohort	Prospective	Unclear	Modified 2002 EORTC/MSG	IPA
Sanguinetti *et al* [*52*]	2003	Italy	Adults with HM	60.3(39–77)	65.0	Cohort	Retrospective	Unclear	Modified 2002 EORTC/MSG	IPA

HM: hematologic malignancy; MTHF: multiple host factors; TR: transplant recipients; IC: immunocompromised; BC: bronchogenic carcinoma; HSCT: hematopoietic stem cell transplant; IPA: invasive pulmonary aspergillosis; 2002 EORTC/MSG13]; 2008 EORTC/MSG[14].

* Mean value in all the included patients.

More characteristics about the included studies can be found in Supplementary Table S1.

Exclusion criteria included: (1) duplicate publications, (2) insufficient data, such as meeting abstracts and conference proceedings, (3) studies with fewer than 20 patients.

**Figure 3:**
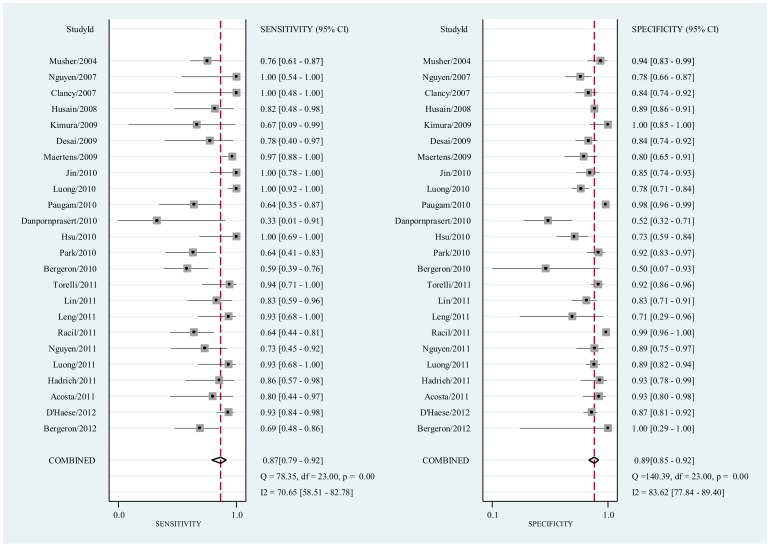
Forest plot of sensitivities and specificities from test accuracy studies of BAL-GM in the diagnosis of IA.

**Figure 4:**
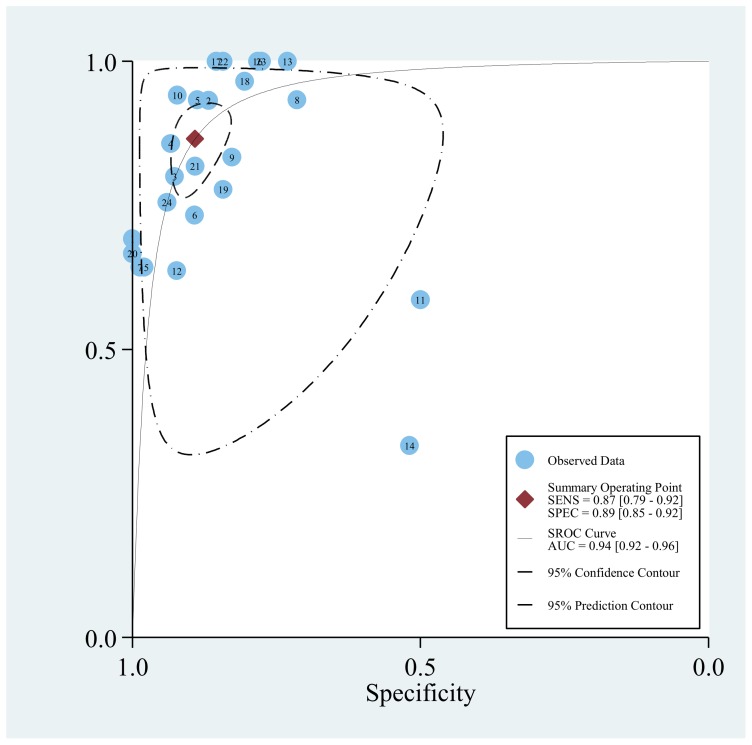
SROC curve for individual studies on the accuracy of diagnosis of IA using BAL-GM. (The correspondence between numbers and the studies can be found in supplement).

### Data extraction and quality assessment

The data was extracted independently by two of the reviewers (Jun Li and Peng Chen), using a pre-designed form, and the information was subsequently entered into Epidata (Odense M, Denmark), or STATA 12.0 (Stata Corp, College station, TX) software. Discrepancies were discussed between investigators and resolved by consensus. For each study, the following information was recorded: first author, year of publication, country or region of origin, ethnicity, mean age, study design, data collection, data for two-by-two tables and so on. Discrepancies between the extracted data were resolved by discussion, and, if required, referred to a third investigator. When the data for a study was not clear and/or not presented by the author in the full-text publications, we contacted the authors for further details. Quality of studies was assessed by using the revised tool for the quality assessment of diagnostic accuracy studies (QUADAS-2) tool[15] and the standards for reporting diagnostic accuracy (STARD) tool[16]. Each item scored a ‘‘yes’’, ‘‘no’’, or ‘‘unclear’’ if there is not sufficient information to make an accurate judgment.

**Figure 5:**
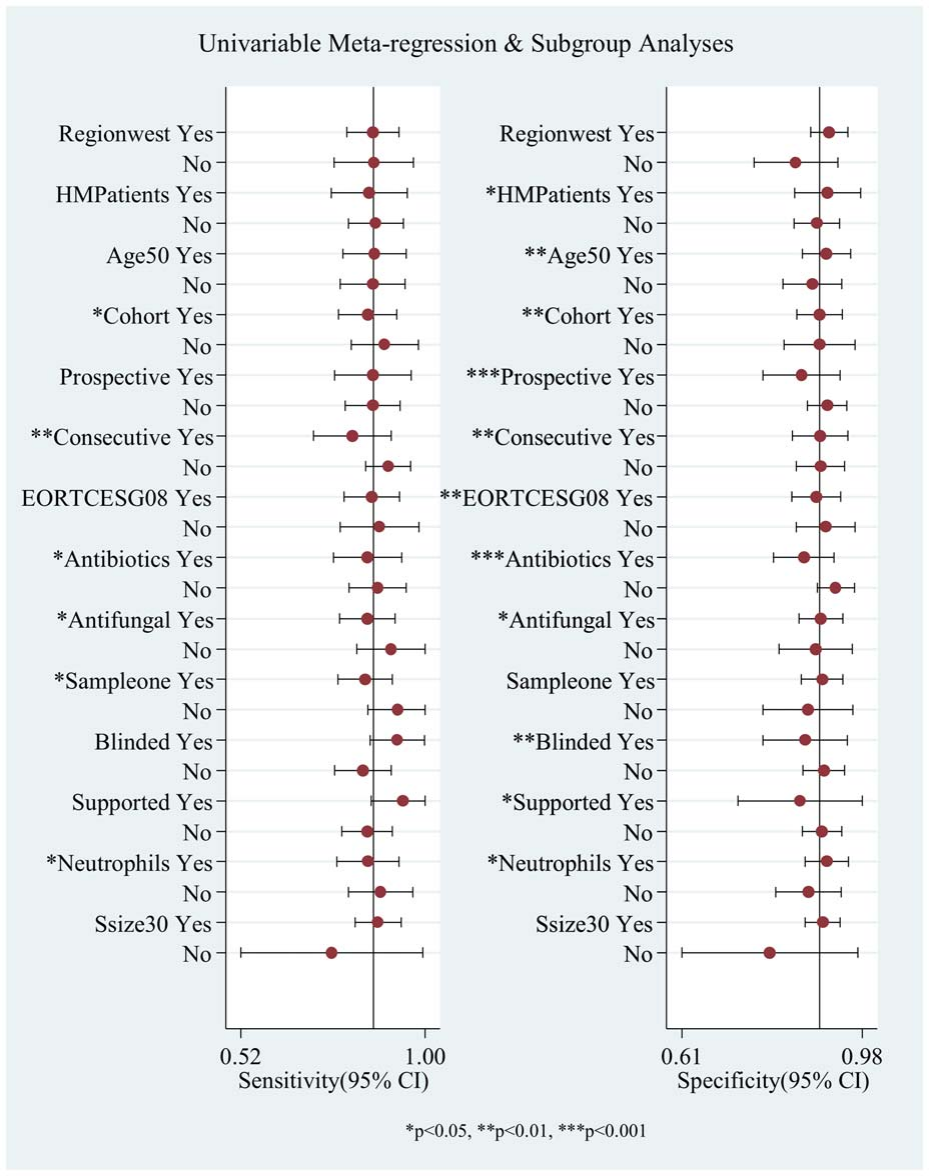
Forest plot of multiple univariable meta-regression and subgroup analyses for SEN and SPE.

**Figure 6:**
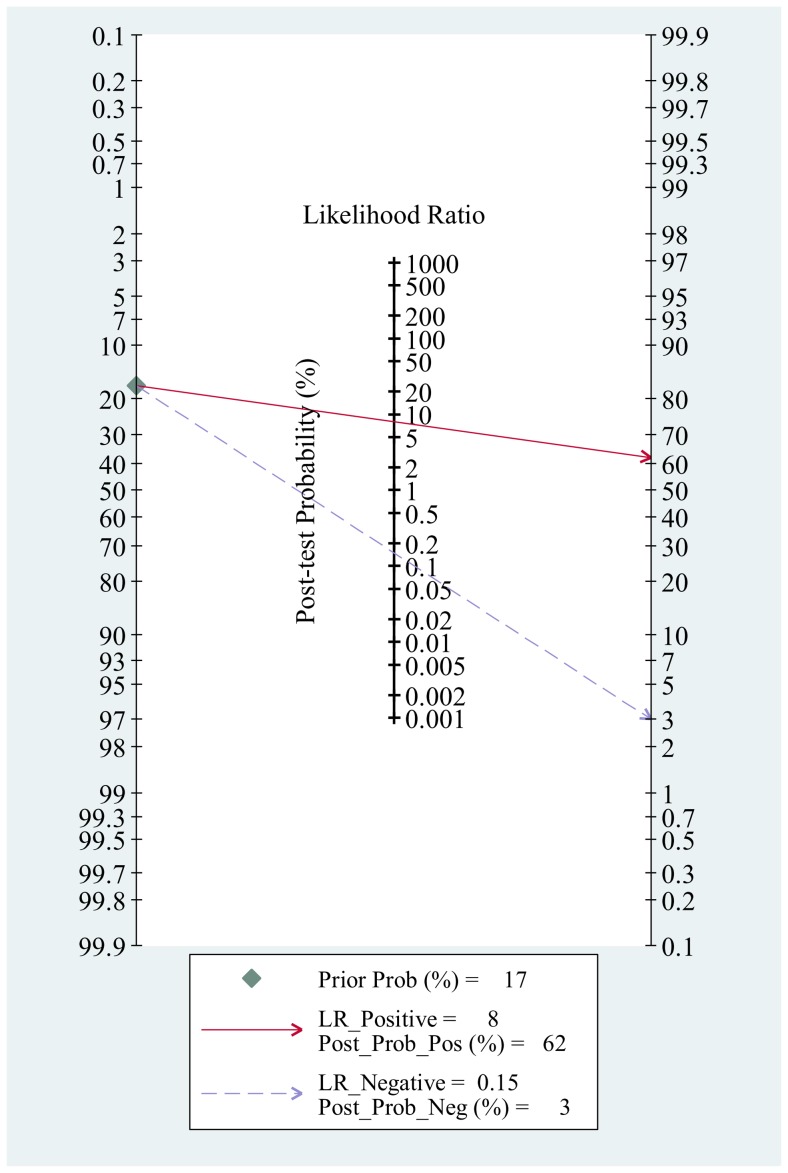
Fagan plot analysis to evaluate the clinical utility of BLAF-GM test.

**Figure 7:**
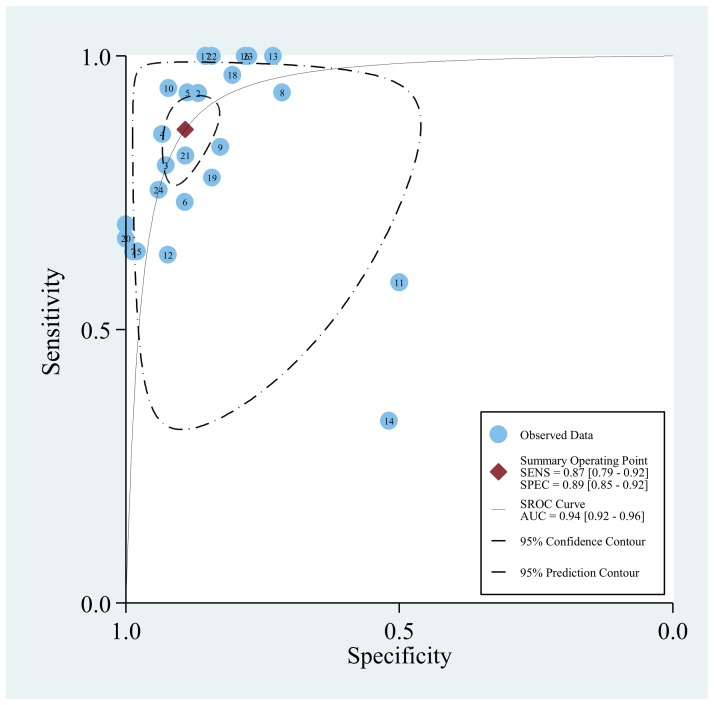
Funnel plot with superimposed regression line.

### Statistical analysis

In this meta-analysis, patients were classified into four groups according to the revised EORTC/MSG: proven IA, probable IA, possible IA, and no IA [14]. For each study, we constructed a two-by-two table cross-classifying BAL-GM test results and the IA ((proven or probable IA vs. possible or no IA) AND (proven IA vs. probable, possible, or no IA)). Because several cutoffs were reported in some studies, we mainly evaluated the cutoff values of 0.5, 1.0, 1.5, 2.0 and 2.5 based on the included studies. As to the studies aiming at comparing *Aspergillus* PCR and BAL-GM test for the diagnosis of IA, we also investigate the pooled SEN and SPE between PCR and BAL-GM by meta-analysis.

**Table 2:** Pooled results of the included studies for IA.

Comparison	Cutoff	Studies	DOR (95% CI)	AUC (95% CI)	SEN*Heterogeneity* (*p*/I^2^)	Pooled SEN (95% CI)	SPE*Heterogeneity* (*p*/I^2^)	Pooled SPE (95% CI)	Pooled PLR (95% CI)	Pooled NLR (95% CI)	Publication bias (*p*)
Proven or probable IA vs. possible or no IA	0.5	24	52.7 (31.8–87.3)	0.94	<0.01/70.65	0.87 (0.79–0.92)	<0.01/83.20	0.89(0.85–0.92)	8.0 (5.7–11.1)	0.15 (0.10–0.23)	<0.01
	1.0	21	112.7 (55.9–227.1)	0.97	<0.01/79.00	0.86 (0.76–0.92)	<0.01/89.04	0.95(0.91–0.97)	17.0 (10.1–28.5)	0.15 (0.09–0.26)	0.21
	1.5	10	143.4 (51.4–400.4)	0.97	<0.01/77.88	0.85 (0.71–0.96)	<0.01/79.41	0.95(0.90–0.97)	17.5 (9.3–32.7)	0.12 (0.04–0.33)	0.61
	2.0	8	97.4 (35.0–270.9)	0.96	<0.01/73.26	0.84 (0.65–0.94)	0.61/0	0.95(0.93–0.96)	16.4 (11.4–23.6)	0.17 (0.07–0.40)	0.66
	2.5	6	79.9 (20.5–311.7)	0.96	<0.01/81.30	0.80 (0.50–0.94)	0.89/0	0.95(0.93–0.97)	16.7 (10.9–25.8)	0.21 (0.07–0.64)	0.56
Proven IA vs. probable, possible, or no IA	0.5	12	8233 (4.7–143631.6)	0.93	<0.01/85.09	1.00 (0.55–1.00)	<0.01/89.17	0.77(0.64–0.86)	4.3 (2.7–6.8)	0.00 (0.00–1.03)	0.06
	1.0	12	168.9 (13.5–2115.6)	0.93	<0.01/77.91	0.97 (0.71–1.00)	<0.01/82.34	0.83(0.75–0.88)	5.6 (3.9–8.2)	0.03 (0.00–0.44)	0.2
	1.5	7	124.3 (13.4–1154.1)	0.97	<0.01/82.50	0.90 (0.42–0.99)	<0.01/74.80	0.93(0.86–0.97)	12.8 (7.1–23.1)	0.01 (0.01–1.02)	1
	2.0	7	50.8 (9.5–271.6)	0.94	<0.01/80.64	0.79 (0.39–0.96)	0.27/21.34	0.93(0.89–0.96)	11.3 (7.0–18.1)	0.22 (0.05–0.91)	0.87
	2.5	5	11.6 (2.4–56.0)	0.93	0.08/51.17	0.45 (0.15–0.79)	0.61/0	0.93(0.90–0.96)	6.9 (2.7–17.3)	0.59 (0.30–1.18)	0.05

As a single indicator measure of the accuracy of a diagnostic test[17], the diagnostic odds ratio (DOR) describes the odds of positive test results in patients with the disease compared with the odds of positive results in those without disease, and corresponds to particular pairings of SEN and SPE [18]. By using a bivariate regression approach, the summary receiver operating characteristic (SROC) curve was constructed to visualize data, and the pooled estimates of SEN and SPE were calculated as the main outcome measures. Meanwhile the summary positive/negative likelihood ratios (pooled PLR and pooled NLR, respectively) were also calculated. A value of pooled PLR greater than 10 and of pooled NLR less than 0.1 were noted as providing convincing diagnostic evidence, while those value more than 5 and less than 0.2 respectively providing strong diagnostic evidence [19], [20]. The between-study heterogeneity was evaluated by the I-square statistic. The DerSimonian Laird method was used for pooled analyses if the value of heterogeneity was more than 50% [21], [22]. To explore the sources of between-study heterogeneity, a meta-regression was used according to the characteristics of the included studies. Subgroup analyses were also performed if necessary. All the analyses mentioned above were conducted in RveMan 5.1 and STATA 12.0 (College Station, TX, USA) with the MIDAS and METANDI modules.

**Figure 8:**
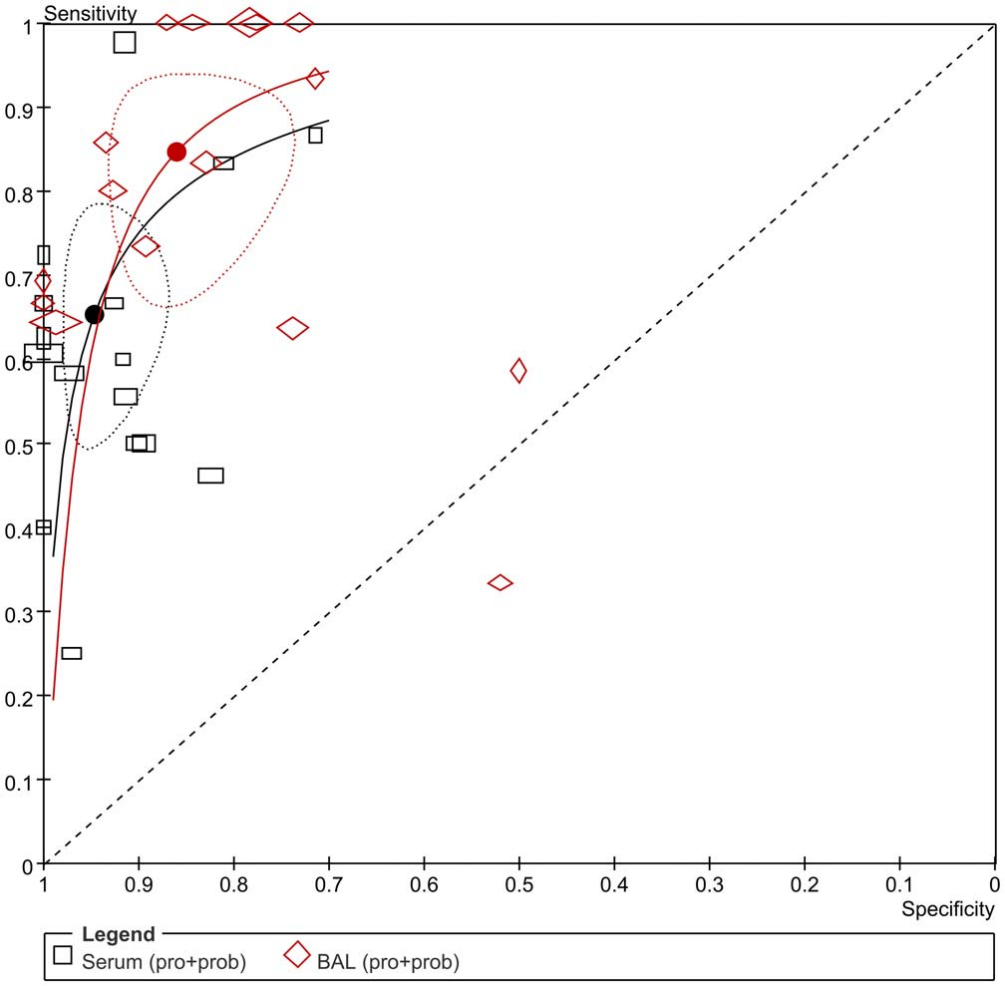
Summary ROC plot of SEN and SPE of serum GM and BAL-GM. (Dotted ellipses around the spots represent the 95% CI around the summary estimates. The diamonds and rectangles represent individual studies and size of the diamonds/rectangles is proportional to the number of patients included in the study).

**Figure 9:**
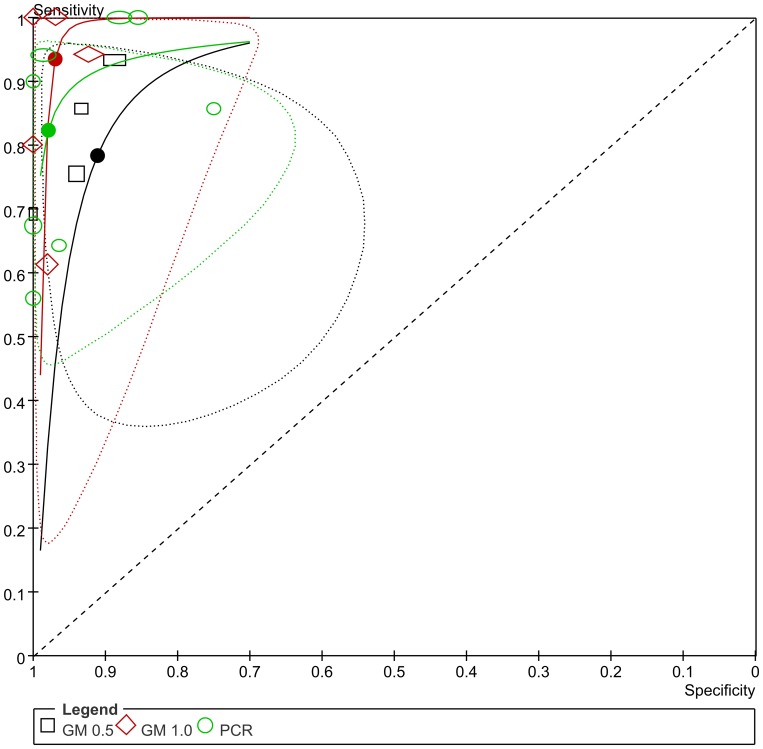
Summary ROC plot of SEN and SPE of PCR and BAL-GM. (Dotted ellipses around the spots represent the 95% CI around the summary estimates. The circles, diamonds and rectangles represent individual studies and the size is proportional to the number of patients included in the study).

## Results

A total of 276 potentially useful relevant articles were initially identified (Figure 1). Then after reviewing the titles by two independent review authors (Zijin Zhao, Luyao Chen), 232 papers were excluded. Furthermore, six studies were excluded after the abstract review (five were not human studies and one was not relevant to BAL-GM test). Eventually, 38 studies were retrieved for further evaluation. According to the inclusion and exclusion criteria, 30 studies[23], [24], [25], [26], [27], [28], [29], [30], [31], [32], [33], [34], [35], [36], [37], [38], [39], [40], [41], [42], [43], [44], [45], [46], [47], [48], [49], [50], [51], [52] were ultimately included for this meta-analysis. The remaining studies were excluded because of lack of sufficient data (n = 3)[53], [54], [55], duplicate publications (n = 2)[56], [57], other diagnosis standard (n = 2)[58], [59] and fewer than 20 patients (n = 1)60].

The main characteristics of the studies included in the meta-analysis are shown in Table 1 and Supplementary Table S1. We included 23 cohort studies and 7 case–control studies. No randomized study was included. A total of 3344 patients or control cases were included, of whom 614 (18.4%) patients were diagnosed with proven or probable IA. The STARD score of each study varied from 10 to 21. The included studies were mainly performed in American, European and Asian countries. Fourteen studies were prospectively designed and seven were case-control studies. The index cutoff of BAL-GM varied from 0.5 to 8.0 in individual studies. The most common value of cutoff was 0.5. Quality assessment is shown with a bar graph according to the QUADAS-2 tool in Figure 2.

### BAL-GM for patients with proven or probable IA

Of all the included studies, 24 studies [23], [24], [25], [26], [27], [28], [29], [30], [31], [32], [33], [34], [35], [36], [37], [39], [40], [41], [42], [44], [47], [48], [49], [50] provided the BAL-GM diagnostic data with a cutoff value of 0.5. Heterogeneity in sensitivities and specificities were observed among the studies (*Q*-test  = 78.35, *P*<0.01, *I^2^* = 0.65% and *Q*-test  = 140.39, *P*<0.01, *I^2^* = 83.20%), which indicated significant heterogeneity for these included studies. The mean DOR was 52.7 (95% CI 31.8–87.3). The pooled SEN was 0.87 (95% CI 0.79–0.92) while the pooled SPE was 0.89 (95% CI 0.85–0.92) (Figure 3, Figure S1). Figure 4 (The corresponding between numbers and the studies could be found in Supplementary Table S2) presents the SROC curve for the including studies. The area under the curve (AUC) was 0.94 (95% CI 0.92–0.96). The pooled PLR and NLR were 8.0 (95% CI 5.7–11.1) and 0.15 (95% CI 0.10–0.23) respectively(Figure S2, S3, S4, S5, and S6).

The proportion of heterogeneity likely due to threshold effect was 44%, which meant a moderate influence of a diagnostic threshold effect. However, the Spearman correlation coefficient was 0.313 and the *P* value was 0.136. To explore other potential heterogeneities, meta-regression and subgroup meta-analysis were conducted (Figure 5). Overall, the test performances varied by patient population, study design and drug treatment. The pooled SEN and SPE were 0.85 (95% CI 0.78–0.93) and 0.89 (95% CI 0.85–0.94) for studies Cohort designed respectively. The pooled SEN of BAL GM test for patients who were given the antibiotic and antifungal treatment were 0.85 (95% CI 0.76–0.94) and 0.85 (95% CI 0.78–0.92), while the pooled SEP were 0.86 (95% CI 0.80–0.92) and 0.89 (95% CI 0.85–0.94) respectively. The pooled SEN changed significantly with some covariates, such as study design(cohort and consecutive), antibiotics using, sample numbers and neutropenia status. The pooled SEP changed significantly with some covariates which are study design(cohort, consecutive, prospective and blinded), patients status(age, hematologic malignancy and neutropenia), treatment(antifungal and antibiotics) and financial support. More detail data is in Supplementary Table S3.

The Fagan plot demonstrated that the BAL-GM test raised the probability of IA from 17% to 62% and decreased the probability to 3% when negative (Figure 6). According to the Deek's funnel plot asymmetry test, the *p* value was less than 0.01 for the slope coefficient, which showed there was a high likelihood of publication bias (Figure 7).

Twenty one [24], [25], [27], [28], [29], [30], [32], [36], [39], [40], [41], [42], [43], [44], [46], [47], [48], [49], [50], [51], ten[24], [25], [30], [36], [39], [41], [44], [48], [49], [52], eight [24], [25], [36], [39], [41], [44], [48], [49] and six [24], [36], [39], [44], [48], [49] studies demonstrated the BAL-GM diagnostic data with a cutoff value of 1.0, 1.5, 2.0 and 2.5 respectively. The mean DOR, pooled SEN, SPE, PLR, NLR and the AUC were summarized in table 2.

### BAL-GM for patients with proven IA

Of the studies that investigated BAL-GM for diagnosing proven IA, Only 12 studies [23], [25], [28], [29], [33], [35], [36], [41], [44], [45], [48], [49] reported the data with a cutoff value of 0.5. The mean DOR was 8233 (95% CI 4.7–143631.6). The pooled SEN and SPE were 1.00 (95% CI 0.55–1.00) and 0.77 (95% CI 0.64–0.86) respectively. The AUC was 0.93. The pooled PLR was 4.3 (95% CI 2.7–6.8) while the pooled NLR was 0.00 (95% CI 0.00–1.03).

The percentage of heterogeneity likely due to threshold effect was 10%, indicating a slight inﬂuence. Meta-regression and subgroup meta-analysis were performed, showing only the study design and diagnostic standard varied the test performances. The pooled SPE, which were lower with those covariates, were 0.67 (95% CI 0.51–0.84) and 0.71(95% CI 0.57–0.85) for prospective studies and studies using the revised EORTC/MSG criteria as gold standard respectively.

The Fagan plot demonstrated that the BAL-GM test raised the probability of IA threefold when results were positive and decreased the probability to 0% when negative. According to the Deek's funnel plot, no publication bias was found (*p* = 0.06, figures not shown).

A few studies investigated the BAL-GM diagnostic data with a cutoff value of 1.0, 1.5, 2.0 and 2.5 respectively. The mean DOR, pooled SEN, SPE, PLR, NLR and the AUC were summarized in table 2.

### Comparison the diagnostic accuracy of serum GM and BAL-GM for patients with IA

Sixteen articles [23], [25], [26], [28], [30], [31], [32], [33], [34], [35], [36], [39], [44], [48], [49], [51] reported both the serum GM and BAL-GM test (cutoff value 0.5) diagnostic data for the proven or probable IA vs. possible or no IA. The pooled SEN of serum GM and BAL-GM test were 0.65 (95% CI 0.54–0.75) and 0.85 (95% CI 0.72–0.92), while the pooled SPE were 0.95 (95% CI 0.90–0.97) and 0.86 (95% CI 0.78–0.92) respectively (Figure 8, Forest plots of SEN and SPE were in additional file). Eight studies [23], [25], [33], [35], [44], [45], [49], [51] demonstrated both diagnostic data for the proven vs. probable or IA possible or no IA(Figure S7, S8, and S9).

### Comparison the diagnostic accuracy of PCR and BAL-GM for patients with IA

Of all the studies included in the review, only eight papers [23], [27], [29], [43], [46], [50], [52], [57] including nine studies evaluated the diagnostic accuracy of PCR and BAL-GM test for prove or probable IA. Four studies [23], [26], [27], [50] reported the BAL-GM diagnostic data with a cutoff value of 0.5 while the others [29], [43], [46], [50], [52] with 1.0. The pooled SEN of BAL-GM (0.5 and 1.0) and PCR were 0.78 (95% CI 0.67–0.87), 0.94 (95% CI 0.68–0.99) and 0.82 (95% CI 0.61–0.93), while the pooled SPE were 0.91 (95% CI 0.84–0.95), 0.97 (95% CI 0.91–0.99) and 0.98 (95% CI 0.85–1.00) respectively(Figure 9, Figure S10).

## Discussion

IA remain a leading cause of morbidity and mortality in immunosuppressed patients[61]. As pulmonary involvement is a hallmark of IA[24], culture or direct microscopic examination of BAL fluid is widely used for evaluation of patients with suspected IA [62]. However, these two methods are limited because they are time-consuming and may produce falsely negative results[11]. Since it is difficult to diagnose IA, many tests have been developed to overcome this problem, including the Platelia GM enzyme immunoassay(Bio-Rad)[10]. Although the kit have been approved by the FDA in 2003 for use with patients with neutropenia and undergoing stem cell transplantation, controversy still exists[1].

To explorer the accuracy of BAL-GM test for diagnosing IA according to the EORTC/MSG definitions or similar criteria, the results of 30 studies were included and analyzed in this meta-analysis. In all, we came to the conclusion that BAL-GM test was an appropriate technique for diagnosing IA, using the cutoff value of 1.0. Compared with GM detection in serum, BAL-GM test has a higher SEN but a lower SPE, and with PCR assay, BAL-GM test has a higher SEN and a similar SPE. Although Guo *et al* [12] have performed a systematic review that evaluated the accuracy of BAL-GM in diagnosing IA, this review included more clinical studies and evaluated the head-to-head comparison of the accuracy of serum GM test, PCR assay and BAL-GM.

Guo *et al* [12], in which proven or probable IA vs. possible or no IA cases were analyzed, performed meta-analysis and obtained a high accuracy, with both the SEN and SPE ≥90%. However, with the different cutoff value, the increasing threshold form 0.5 to 2 decreased the pooled SEN from 0.86 to 0.61, and increased the pooled SPE from 0.89 to 0.96. Comparing with the pooled SEN and SPE in Guo's study, this current meta-analysis obtained a similar SEN and SPE with the cutoff value of 0.5 and 1.0, but a higher SEN and similar SPE with the cutoff value of 1.5 and 2.0, in which the pooled SEN and SPE were from 0.87 to 0.84 and 0.89 to 0.95 respectively. This higher SEN may have resulted from more studies included. Likelihood ratios are also investigated as a metric that incorporate both the SEN and SPE in this systematic review. It has been suggested that a PLR more than 10 and NLR less than 0.1 provides convincing diagnostic evidence, and a PLR more than 5 and NLR less than 0.2 provides strong diagnostic evidence to rule in/rule out diagnoses respectively in most circumstances[20], [63]. The conclusion of Guo's meta-analysis showed that the PLR and NLR succeeded in passing the threshold index and provided convincing diagnostic evidence to rule in/rule out IA with the result of overall analyses. However no results of meta-analyses with different cutoff values passed. Our meta-analyses got the similar PLR and NLR with Guo's, but only strong diagnostic evidence was suggested based on results of individual meta-analysis. Apart from SEN, SPE, AUC, PLR and NLR, we also reported another indicator of test performance, which is DOR. The DOR combines the strengths of SEN and SPE and has the advantage of accuracy as a single indicator[17]. Not only are the DORs estimated by classic meta-analytic approach, but also DORs are produced by bivariate approach. Bivariate approach was used in this meta-analysis because it maintains any correlation between SEN and SPE, while conventional meta-analysis splits the assessment of these at study level[18]. The DOR varied from 52.7 to 143.4 with different cutoff values, which were all high. According to results mentioned above, the optimal cutoff value was not 0.5 but 1.0, because, compared to 0.5, it has higher DOR, SPE and PLR, and similar SEN and NLR.

Serum GM has been approved by FDA for diagnosing IA[64], and meta-analysis found it was moderately useful for surveillance of IA in patients with hematological malignancy or hematological transplant recipients[65]. Studies showed that BAL-GM test was superior than serum GM test, however, no direct meta-analysis of comparison of serum GM and BAL-GM has been done. This study firstly performed comparison of serum GM and BAL-GM test by meta-analysis, and the results showed that, for proven and probable IA, the pooled SEN and SPE of serum GM were 0.65 (95% CI 0.54–0.75) and 0.95 (95% CI 0.90–0.97) respectively. The results of summary estimates of serum GM were similar to the meta-analysis conducted by Pfeiffer *et al* [65]. Compared with serum GM, BAL-GM has a higher SEN [0.85 (95% CI 0.72–0.92)] and lower SPE [0.86 (95% CI 0.78–0.92)]. The higher SEN of BAL-GM test may have two reasons. One is that the bronchial tree of patients with pulmonary IA, which is the most common presentation of IA, has a larger fungal burden. The other one is that hyphae secrete more quantities of antigenic GM than conidia[42], [56]. The lower SPE may result from that the airway and vascular compartments are involved in different stages of disease[28]. Studies have showed that the appearance of GM in the BAL fluid correlated with the airway cellular invasion of *Aspergillus* [66], while the presence of GM in serum correlates with the later penetration of hyphae through the endothelial cell layer[66], [67]. So it is suggested that BAL-GM and serum GM testing are complementary based on the our meta-analysis.

PCR assay for the detection of fungal nucleic acids in BAL fluid was investigated. Studies indicated that PCR had variable SEN which ranged from 40 to 100% [23], [26], [27], [29], [43], [46], [50], [52]. The variety may be due to differences in assay characteristics, certainty of diagnosis and types of patients evaluated[50]. More and more studies evaluated PCR on BAL fluid for diagnosing IA, however lack of standard assay platform hampered its wide use. To our knowledge, this systematic review is also the first study which conducted meta-analysis of comparison of PCR assay and BAL-GM test for diagnosing IA. In contrast to BAL-GM with the cutoff value of 0.5, PCR has a slight higher SEN and a significant higher SPE. Compared with BAL-GM (cutoff value 1.0), PCR displays a lower SEN and a similar SPE. One of questionable points in this part of study are the increasing threshold of BAL-GM test from 0.5 to 1.0 increased the SEN from 0.78 to 0.94. It may be because of study designed, type of patients evaluated or other biases. So more high quality, well-designed studies are needed to estimate the comparison between PCR assay and BAL-GM test for diagnosing IA.

This current study shows that BAL-GM has a better capacity for diagnosing IA than both serum GM test and PCR assay test, but it has its own inherent limitations. The high SEN of BAL-GM might be counterbalanced by the occurrence of false positive results[45]. False negativity has been reported in several studies[34], [41], [54] and is a major drawback of this technique[54]. Firstly, the β-lactam antibiotics such as amoxicillin-clavulanate and piperacillin-tazobactam, which are likely to be given to the patients, have been reported to caused false positive results at different rates[54]. Secondly, it is reported that some fungi contained cross-reactive GM[68], [69]. Last but not least, some other factors such as antifungal prophylaxis, airway colonization with *Aspergillus* species and even laboratory contamination may result in false positive results[41]. So physicians should be aware of the false positive results mentioned above when interpreting GM results.

There are several limitations to our study. First, significant heterogeneity existed in most of the analyses. To investigate the sources of heterogeneity, sensitivity, subgroup and meta-regression analyses were performed. Sensitivity analyses were conducted after deleting the studies with outlier results[30], [33], [36], [37], [44], however, the heterogeneity still exist and the pooled results has slight changes. The subgroup and meta-regression analyses found some study characteristics including patients status, age, study design, reference criteria, antibiotic and antifungal treatment that account for the heterogeneity. The difference in patient status had statistical significance for the SEN and the difference in age, study design and reference criteria had statistical significance for the SPE. Despite this, most of the pooled SEN and SPE were still above 85%, indicating that BAL-GM test has excellent accuracy. Secondly, although we search the studies published in any languages, we didn't search for unpublished data. Diagnostic studies are easy to undertake and are not usually recorded on research registries, so it is difficult for researchers to search for unpublished data[70]. Therefore, some missing and unpublished data might not have been included in the current study, which may have overestimated the pooled results. Thirdly, misclassification bias can occur[12]. At present, the gold standard for the diagnosis of *Aspergillus* infection is isolation and culture of the organisms in the laboratory[71], but it is limited by complications and low SEN. According to the reference criteria which most of included studies used, the proven and probable IA were not diagnosed by either cytopathologic and histopathologic examination. So it is unavoidable that the accuracy of diagnosis cause misclassification and discrepancy, which resulted in biased results.

## Conclusions

In summary, despite the limitations mentioned above, this current systematic review suggests that the BAL-GM test is a useful adjunct in the diagnosis of IA and the optimal cutoff value is 1.0. The BAL-GM test has higher SEN compared to PCR and serum GM test with the cutoff value of 1.0.

## Supporting Information

Figure S1 Paired forest plot depiction of empirical Bayes predicted versus observed sensitivity and specificity.
(TIF)

Figure S2 Graphical depiction of residual-based goodness-of-fit, bivariate normality, inﬂuence and outlier detection analyses.
(TIF)

Figure S3 Bivariate box plot.
(TIF)

Figure S4 Hierarchical summary ROC curve with confidence and prediction regions around mean operating sensitivity and specificity point.
(TIF)

Figure S5 Probability Modifying Plot.
(TIF)

Figure S6 Likelihood ratio scattergram.
(TIF)

Figure S7 Forest plots of sensitivity and specificity of serum GM and BAL-GM test for diagnosing proven or probable Invasive *Aspergillosis*.
(TIF)

Figure S8 Forest plots of sensitivity and specificity of serum GM and BAL-GM test for diagnosing proven Invasive *Aspergillosis*.
(TIF)

Figure S9 The summary ROC curve of serum GM and BAL-GM test for diagnosing proven Invasive *Aspergillosis*.
(TIF)

Figure S10 Forest plots of sensitivity and specificity of PCR assay and BAL-GM test for diagnosing proven or probable Invasive *Aspergillosis*.
(TIF)

Table S1 Detail characteristics of studies included in the Meta-analysis of diagnosis of IA using BAL-GM.
(XLSX)

Table S2 The correspondence between numbers and the studies.
(XLSX)

Table S3 Detail information of multiple univariable meta-regression and subgroup analyses.
(XLSX)
