# Correction: Systematic Review and Meta-Analysis of Detecting Galactomannan in Bronchoalveolar Lavage Fluid for Diagnosing Invasive *Aspergillosis*

**DOI:** 10.1371/journal.pone.0190459

**Published:** 2017-12-27

**Authors:** Mingxiang Zou, Lanhua Tang, Shushan Zhao, Zijin Zhao, Luyao Chen, Peng Chen, Zebing Huang, Jun Li, Lizhang Chen, Xuegong Fan

Fig 7 is incorrect. The authors have provided a corrected version here.

**Fig 7:**
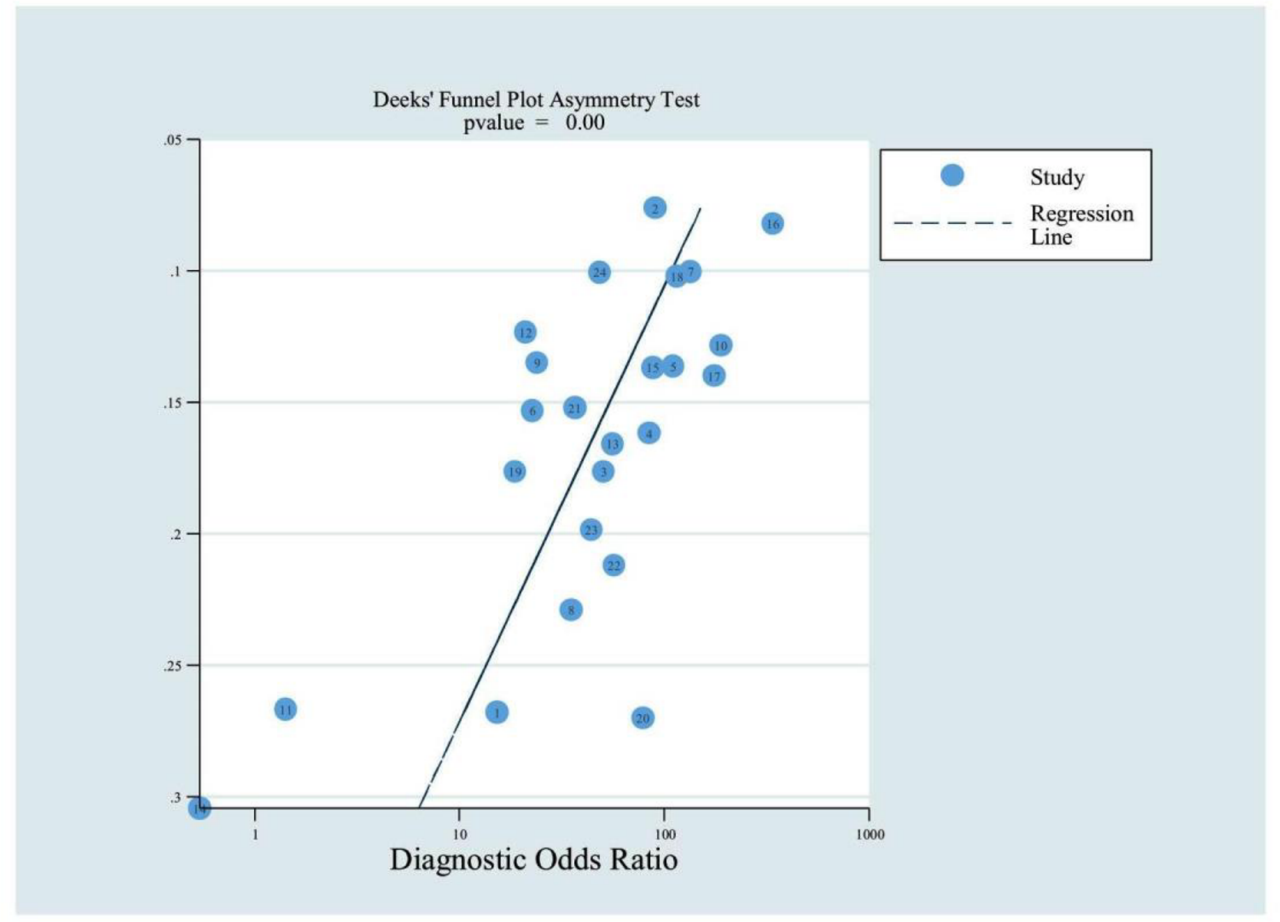
Funnel plot with superimposed regression line.
