# Supplementary figures and images for: Systematic Review and Meta-Analysis of Detecting Galactomannan in Bronchoalveolar Lavage Fluid for Diagnosing Invasive Aspergillosis
Source: PLoS One. 2012 Aug 14;7(8):e43347. doi: 10.1371/journal.pone.0043347 (PMC3419176; doi:10.1371/journal.pone.0043347)

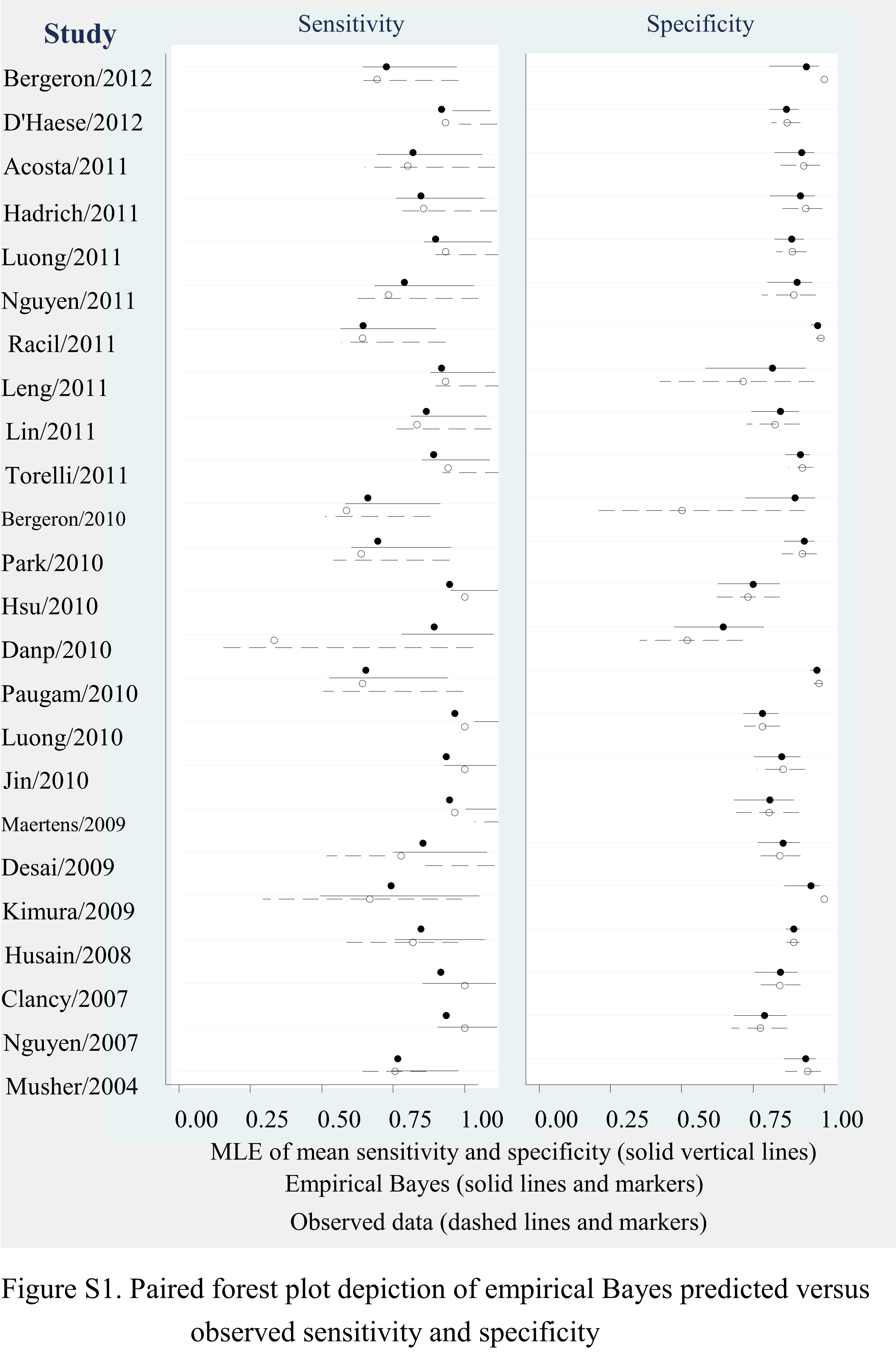

Supplement: Figure S1 — Paired forest plot depiction of empirical Bayes predicted versus observed sensitivity and specificity. (TIF) [file pone.0043347.s001.tif]

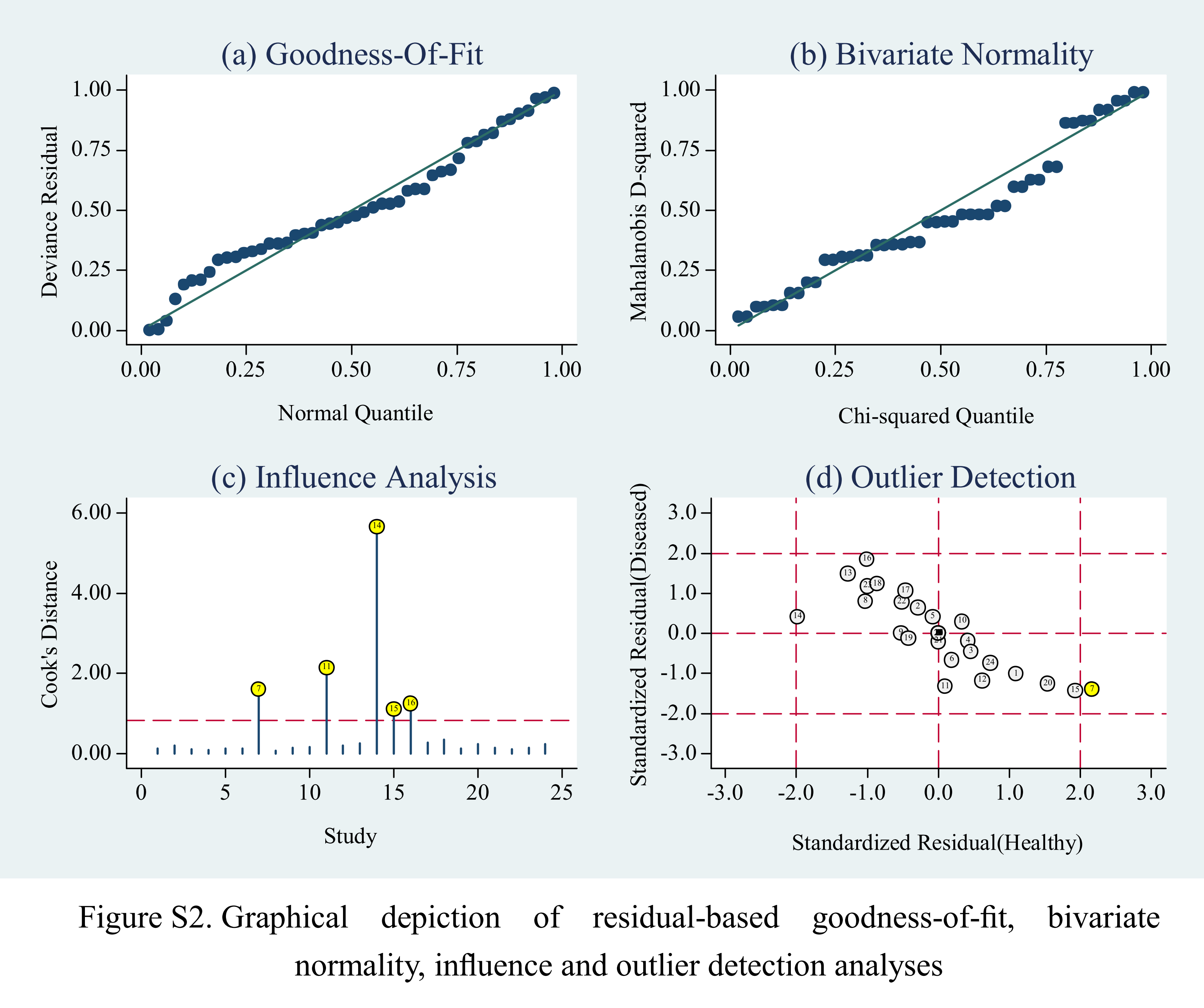

Supplement: Figure S2 — Graphical depiction of residual-based goodness-of-fit, bivariate normality, inﬂuence and outlier detection analyses. (TIF) [file pone.0043347.s002.tif]

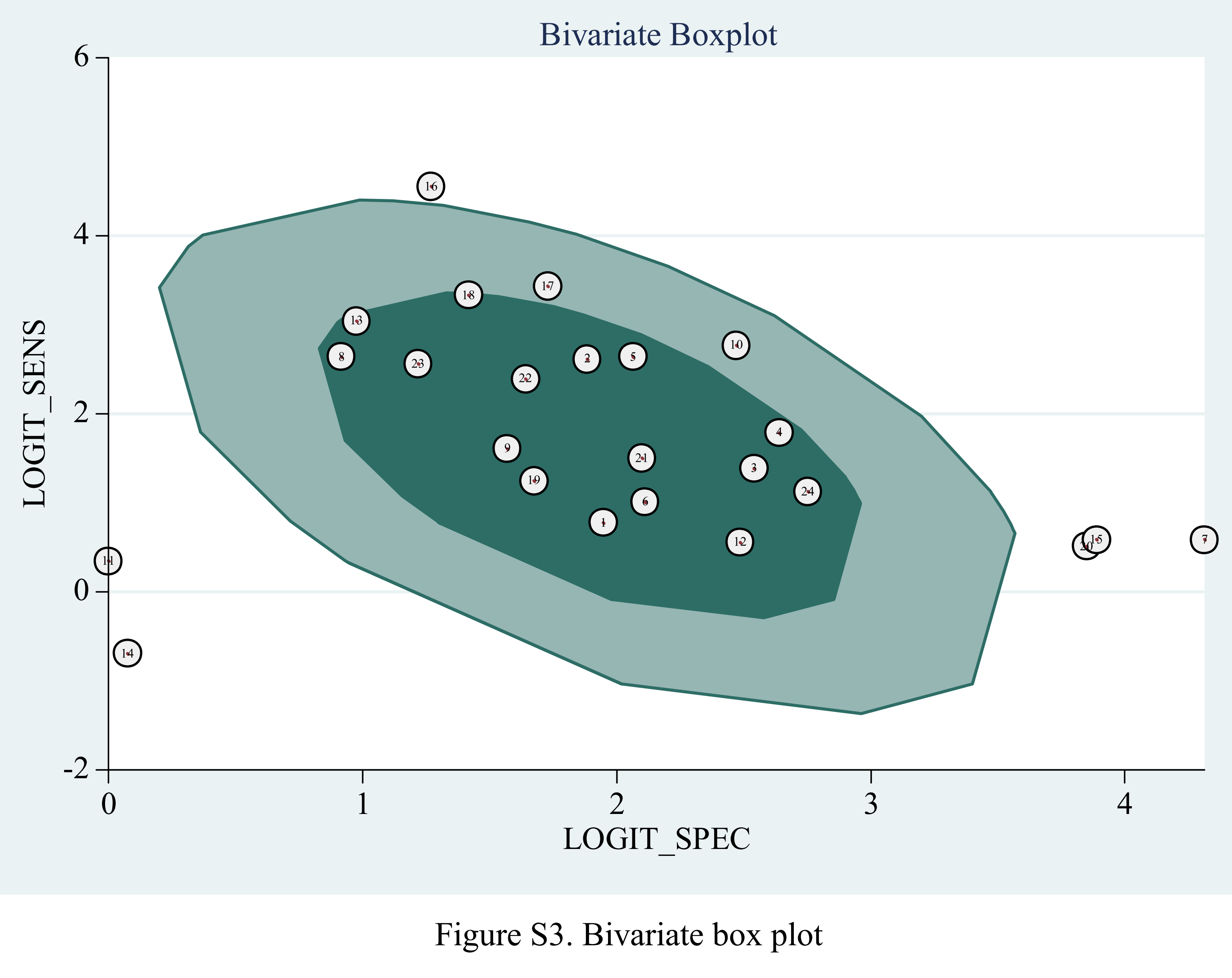

Supplement: Figure S3 — Bivariate box plot. (TIF) [file pone.0043347.s003.tif]

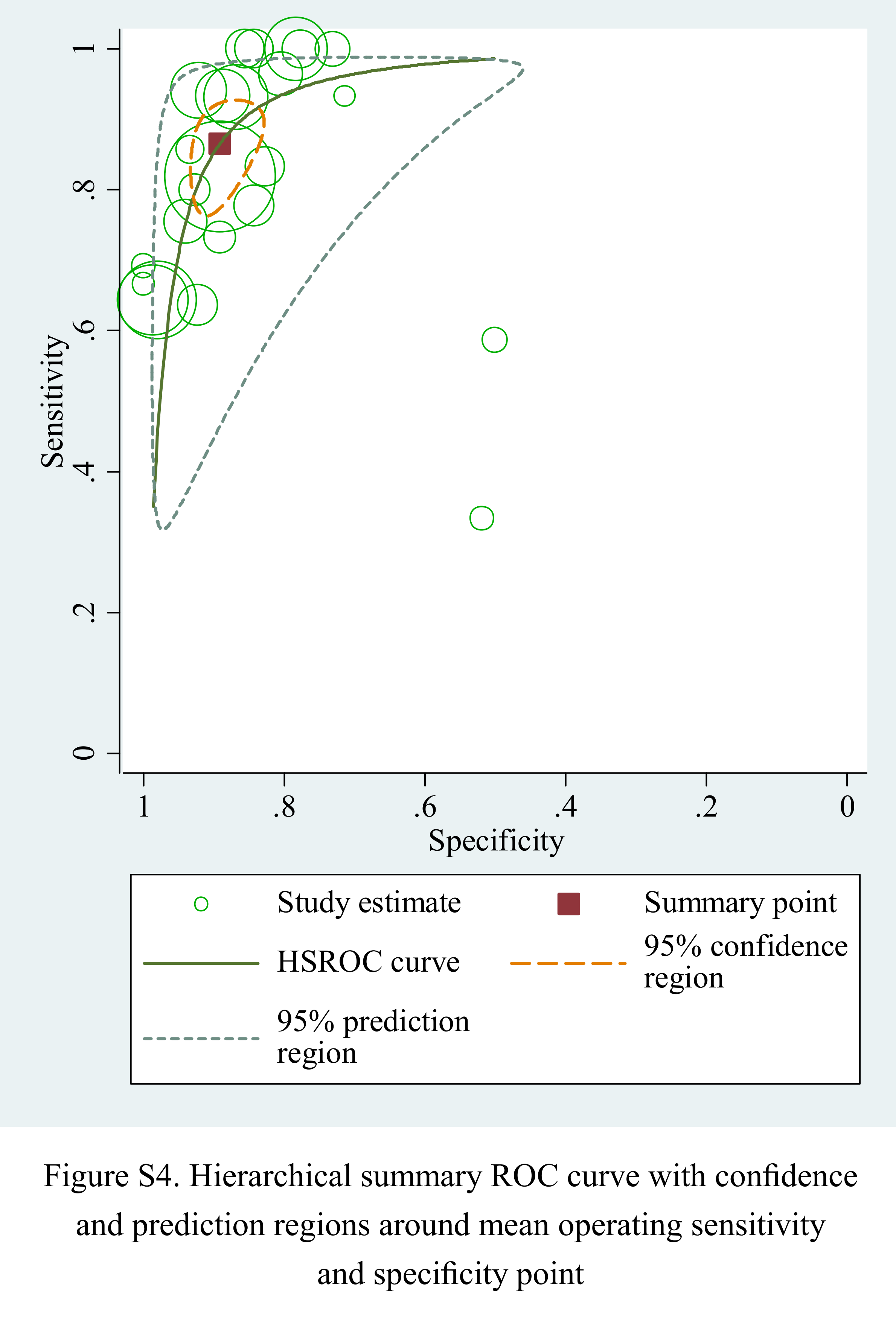

Supplement: Figure S4 — Hierarchical summary ROC curve with confidence and prediction regions around mean operating sensitivity and specificity point. (TIF) [file pone.0043347.s004.tif]

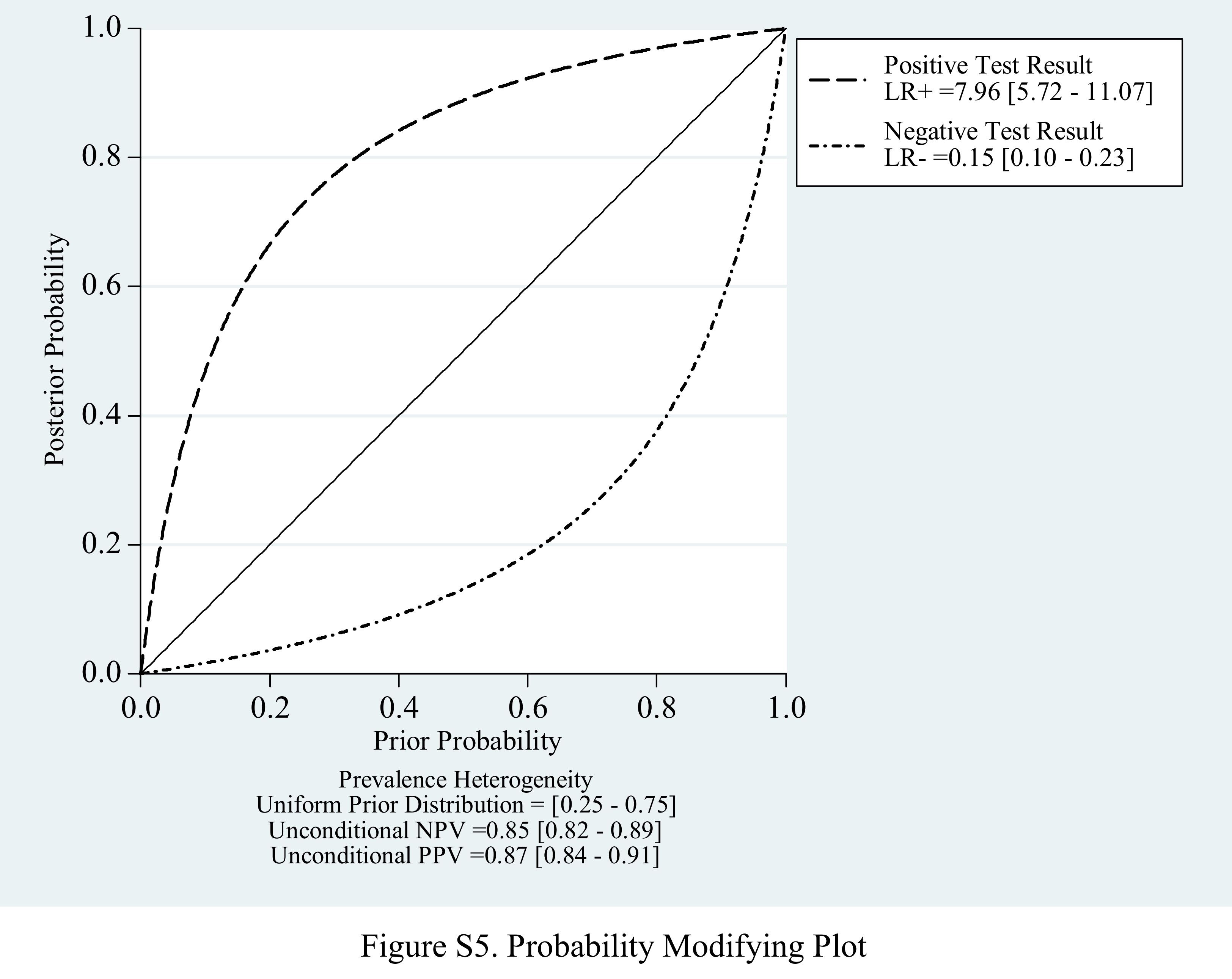

Supplement: Figure S5 — Probability Modifying Plot. (TIF) [file pone.0043347.s005.tif]

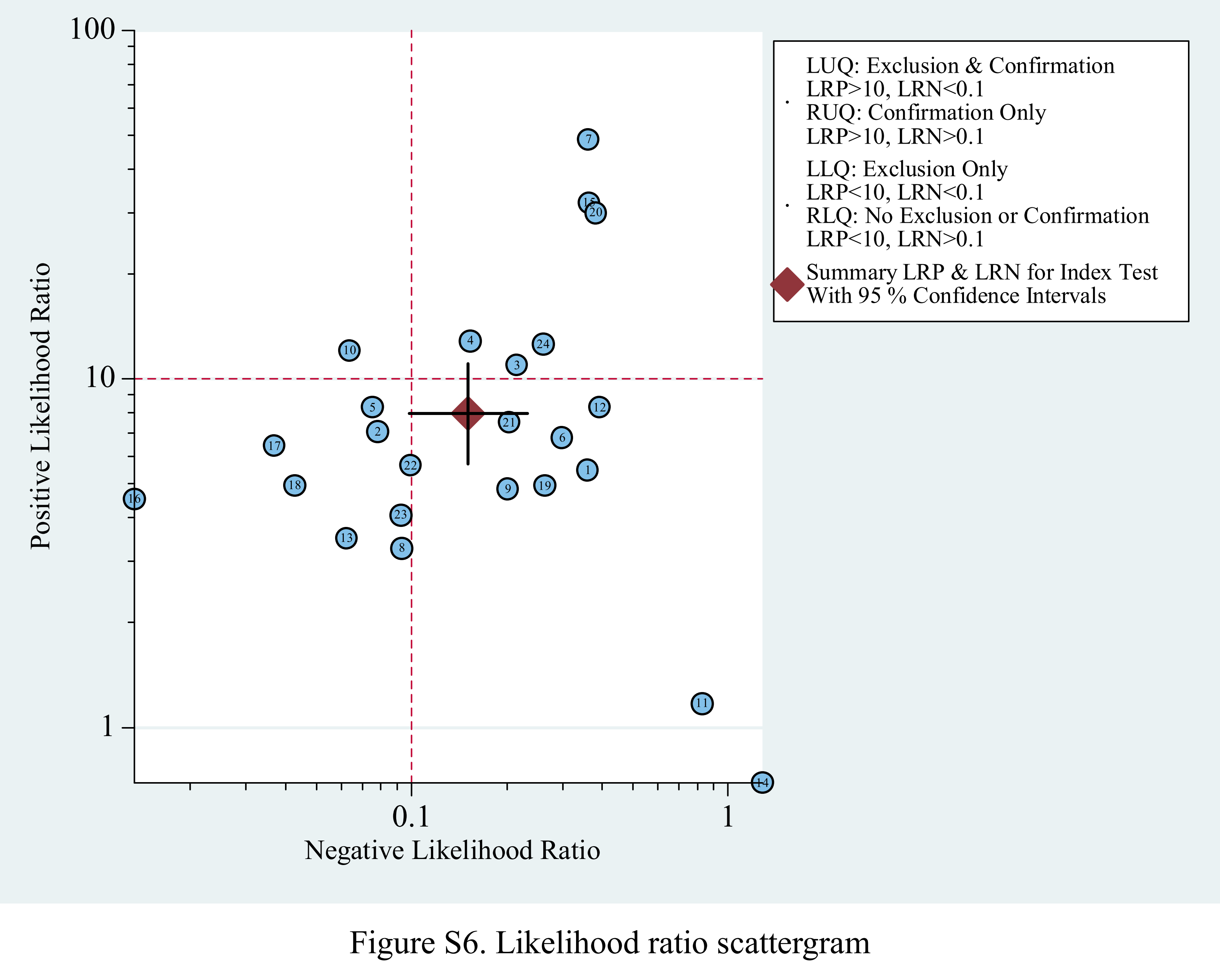

Supplement: Figure S6 — Likelihood ratio scattergram. (TIF) [file pone.0043347.s006.tif]

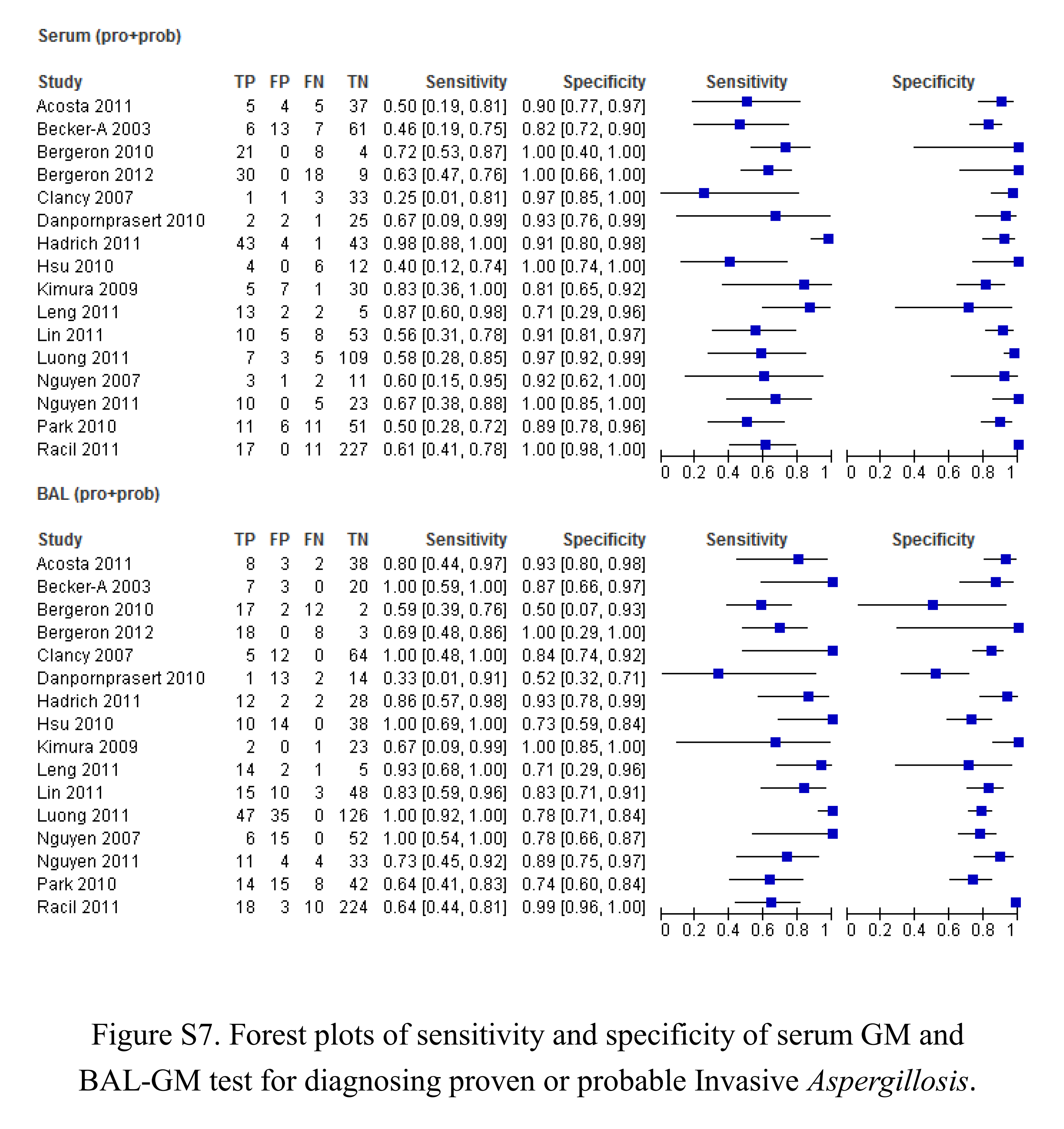

Supplement: Figure S7 — Forest plots of sensitivity and specificity of serum GM and BAL-GM test for diagnosing proven or probable Invasive Aspergillosis. (TIF) [file pone.0043347.s007.tif]

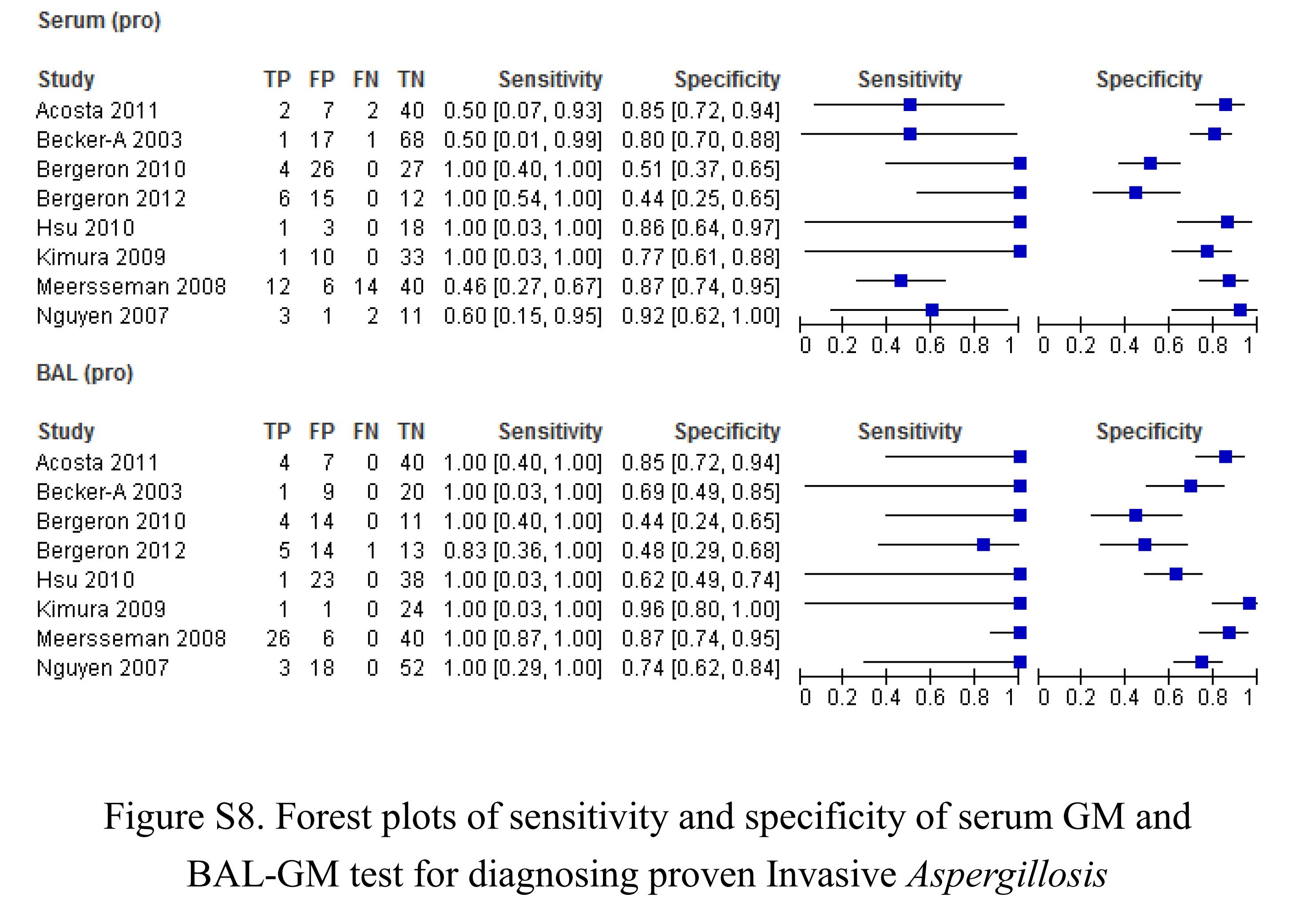

Supplement: Figure S8 — Forest plots of sensitivity and specificity of serum GM and BAL-GM test for diagnosing proven Invasive Aspergillosis. (TIF) [file pone.0043347.s008.tif]

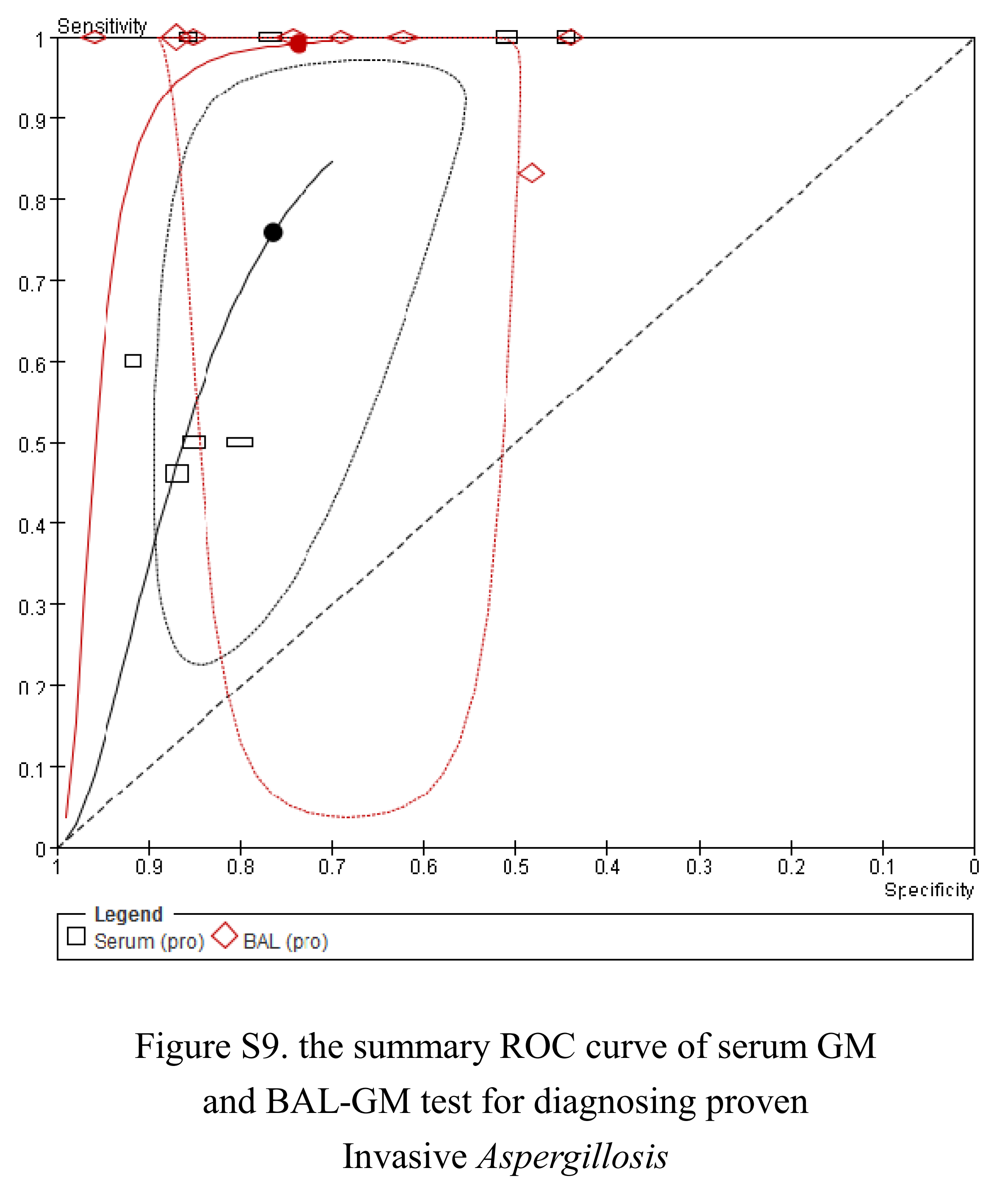

Supplement: Figure S9 — The summary ROC curve of serum GM and BAL-GM test for diagnosing proven Invasive Aspergillosis. (TIF) [file pone.0043347.s009.tif]

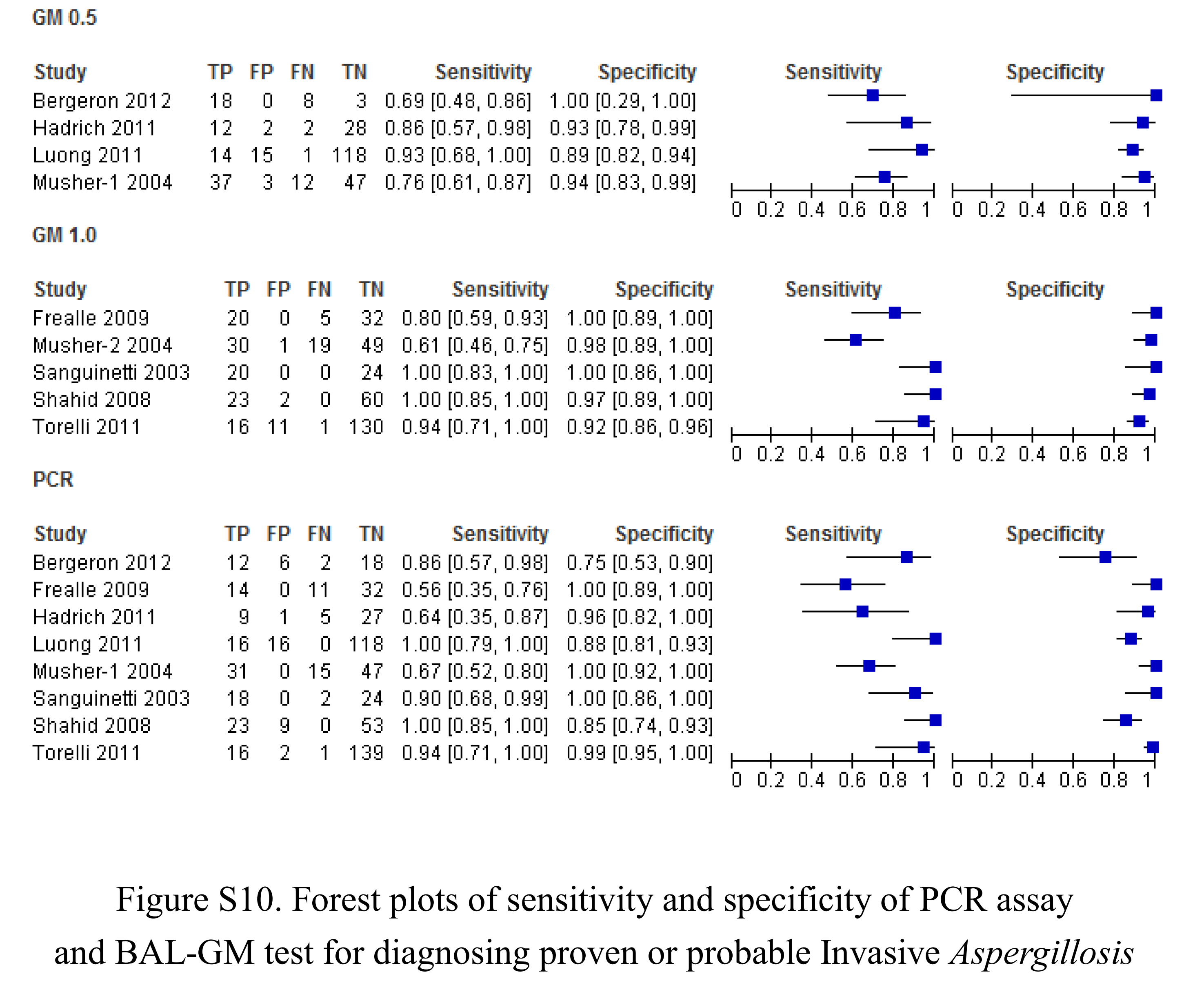

Supplement: Figure S10 — Forest plots of sensitivity and specificity of PCR assay and BAL-GM test for diagnosing proven or probable Invasive Aspergillosis. (TIF) [file pone.0043347.s010.tif]
